# Spindle cell sarcoma of the right atrium causing right atrial pseudoaneurysm: a case report and review of the literature

**DOI:** 10.1186/s13019-021-01404-2

**Published:** 2021-03-19

**Authors:** Xiaofang Wang, Aiqiang Dong, Weijun Yang, Qunjun Duan

**Affiliations:** Department of Cardiovascular Surgery, The Second Affiliated Hospital of Zhejiang University School of Medicine, No. 88 Jiefang Road, Hangzhou, China

**Keywords:** Spindle cell sarcoma, Right atrium, Pseudoaneurysm

## Abstract

**Background:**

Spindle cell sarcomas of the right atrium are extremely rare primary cardiac tumours, with very few cases reported in the medical literature. Pseudoaneurysms caused by cardiac spindle cell sarcoma have never been reported worldwide.

**Case presentation:**

A 32-year-old woman was referred to our hospital for recurrent pericardial haemorrhagic effusion and pleural effusion. Three-dimensional transthoracic echocardiogram, contrast chest CT, and contrast MRI revealed a pseudoaneurysm on the right side of the right atrium with a thrombus. There was a defect between the pseudoaneurysm and the right atrium. PET-CT suggested that FDG metabolism inhomogeneity increased in the mass in the right atrium. Exfoliative cytology detection of massive pericardial effusion and pleural effusion revealed no tumour cells. Spindle cell sarcoma of the right atrium was not confirmed until the patient underwent right thoracic exploration and biopsy. Before a confirmed diagnosis, symptomatic treatment, such as chest effusion and pericardium effusion drainage, and transfusion of red blood cells were mainly used to relieve the patient’s symptoms. Unfortunately, the patient was lost to optimal treatments and passed away 20 days after the pathological diagnosis was made.

**Conclusion:**

The prognosis of spindle cell sarcomas remains poor due to delays in diagnosis, early metastasis and few available therapeutic options. Recurrent pericardial effusion and pleural effusion, especially in the nature of haemorrhagic effusion, and/or right atrial pseudoaneurysm shown on the transthoracic echocardiogram must be considered and highly suspected as malignancy by patients and physicians. If the diagnosis cannot be confirmed, histopathology should be performed as soon as possible to avoid losing the best treatment opportunity.

**Supplementary Information:**

The online version contains supplementary material available at 10.1186/s13019-021-01404-2.

## Introduction

Primary cardiac tumours are rare, with a reported incidence of 0.017 to 0.019% [1]. Only 25% are malignant, with 95% of these being reported as sarcomas. The most common sarcomas are angiosarcoma (34%) and undifferentiated sarcoma (24%). Others include rhabdomyosarcoma, osteosarcoma, synovial sarcoma and leiomyosarcoma [1, 2]. Spindle cell sarcoma, classified as undifferentiated sarcoma, also known as intimal sarcoma, is the least reported. It is characterized by a difficult diagnosis, early metastasis, and a poor prognosis because of the lack of a gold-standard noninvasive examination, high malignancy and a lack of effective therapy [3, 4]. Symptoms are atypical, such as embolism, obstruction, and local invasion. The following case is unique in that spindle cell sarcoma of the right atrium caused right atrial pseudoaneurysm, and to the best of our knowledge, such a case has never been reported worldwide.

## Case presentation

A 32-year-old previously healthy woman initially sought medical advice in a primary hospital in September 2019 with a chief complaint of repeated shortness of breath, vomiting and diarrhoea. Transthoracic echocardiogram suggested pericardial effusion without any suspicious mass in the image. Pericardial puncture drained haemorrhagic fluid. A chest computed tomography (CT) scan revealed bilateral pleural effusion without any visual mass in the image. Thoracic puncture drained haemorrhagic fluid (left thoracic cavity) and light-yellow fluid (right thoracic cavity). Pericardial biopsy showed nonspecific pericarditis. Tuberculosis was excluded from a negative T-spot test, tuberculin test and sputum smear. Finally, the patient was diagnosed with nonspecific pericarditis. She underwent pericardial effusion drainage and pleural effusion drainage. The patient was discharged from the hospital after symptom relief.

In March 2020, the patient was admitted to our institution with shortness of breath and pain of the right shoulder. Physical examination revealed a marked pale appearance, distant heart sounds without a heart murmur, and weak breath sounds in the right lung. She had no fever and no significant weight loss. The laboratory tests showed notable decreases in haemoglobin and albumin, with other blood tests being normal. No tumour marker was elevated. Exfoliative cytology detection of massive pleural effusion and pericardial effusion revealed no tumour cells.

**Fig. 1:**
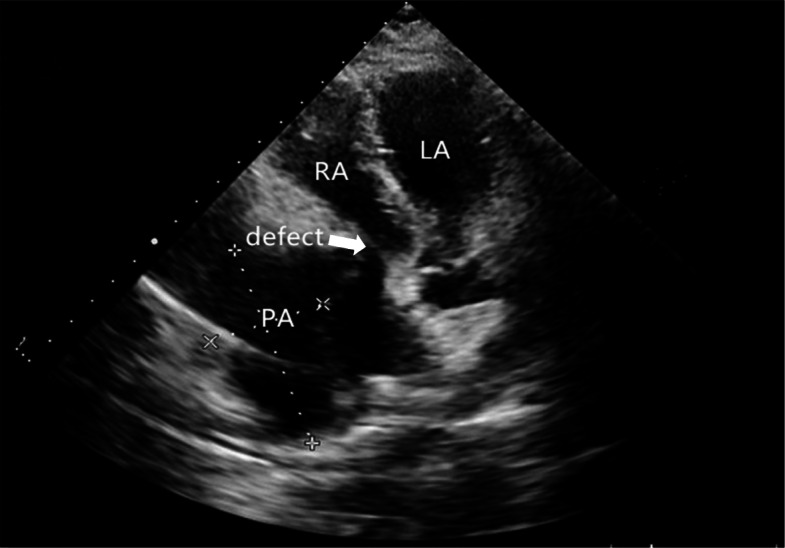
Three-dimensional transthoracic echocardiogram. TTE shows an irregular cystic mass with a pseudoaneurysm measuring 8.49*3.81 cm in size in the lateral wall of the right atrium, and the mass obviously compressed the right atrium. A defect was observed between the pseudoaneurysm and the right atrium. LA: left atrium; RA: right atrium; PA: pseudoaneurysm

**Fig. 2:**
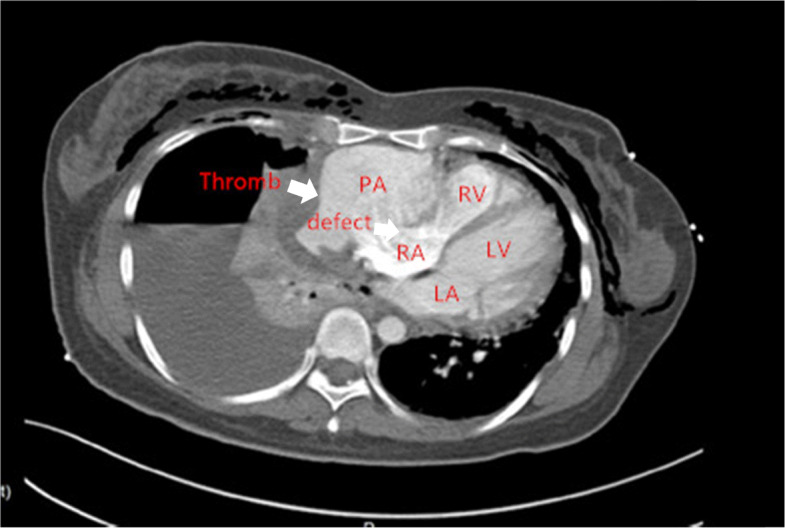
CT. A contrast chest CT scan displays a pseudoaneurysm with a thrombosis inside the right atrium. Thromb: thrombosis; PA: pseudoaneurysm; RA: right atrium; RV: right ventricle; LA: left atrium; LV: left ventricle

**Fig. 3:**
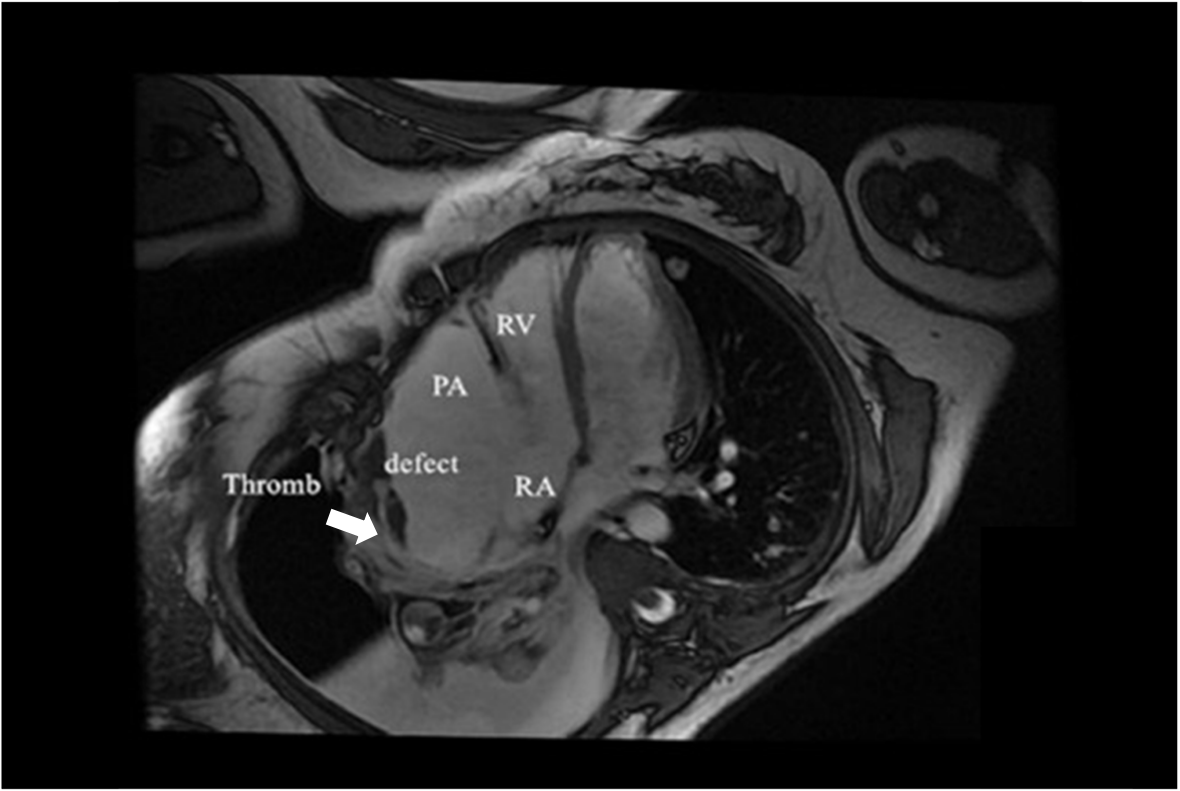
MRI. Contrast MRI revealed a pseudoaneurysm measuring 100 mm*48 mm on the right side of the right atrium with a thrombus. There was a defect measuring 22.5 mm between the pseudoaneurysm and the right atrium. Thromb: thrombosis; PA: pseudoaneurysm; RA: right atrium; RV: right ventricle

Three-dimensional transthoracic echocardiogram (TTE) showed an irregular cystic mass measuring 8.49*3.81 cm in the lateral wall of the right atrium, indistinctly demarcated from the pericardium. The mass obviously compressed the right atrium (Fig. 1). A contrast chest CT scan displayed a pseudoaneurysm with thrombosis inside the right atrium (Fig. 2). Multiple nodules in both lungs and on the right pleura, as well as right hydropneumothorax, were also noted. Contrast MRI demonstrated a 100 mm*48 mm pseudoaneurysm on the right side of the right atrium with a thrombus. There was a defect measuring 22.5 mm between the pseudoaneurysm and the right atrium (Fig. 3). PET-CT suggested that FDG metabolism inhomogeneity increased in the mass in the right atrium. FDG metabolism slightly increased in multiple nodules in both lungs. Multiple ground glass foci in both lungs were considered bleeding.

**Fig. 4:**
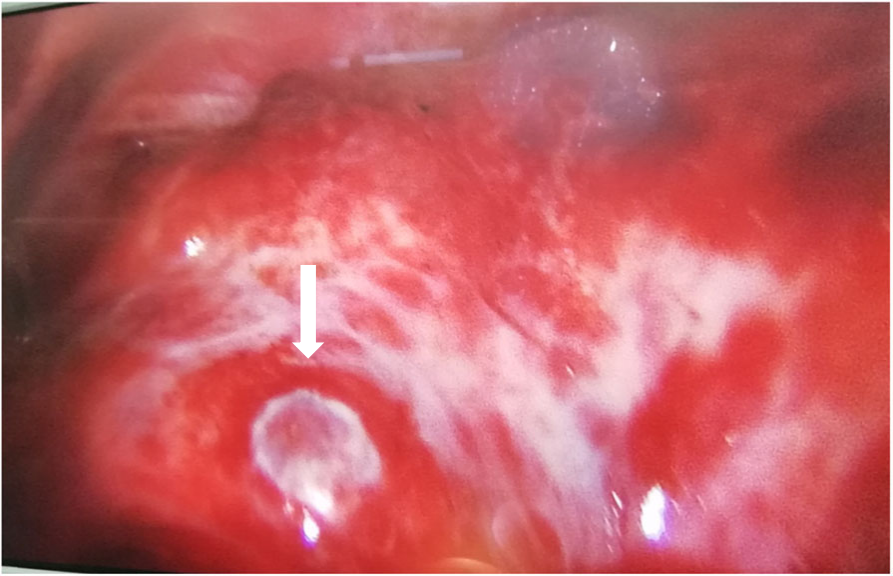
Surgical inspection. A massive lobulated and friable tumour in the pericardium with severe bleeding

**Fig. 5:**
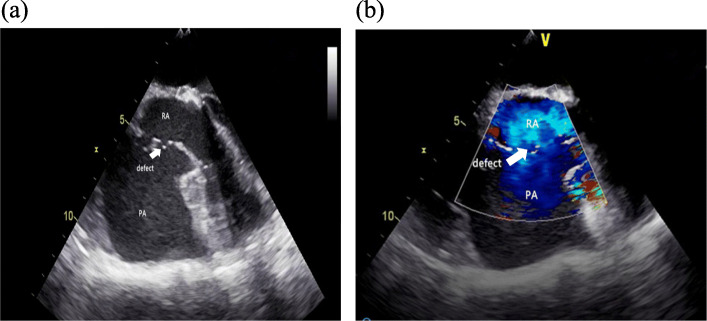
Intraoperative TEE. Intraoperative transoesophageal echocardiography shows right atrium rupture with pseudoaneurysm formation. PA: pseudoaneurysm; RA: right atrium

**Fig. 6:**
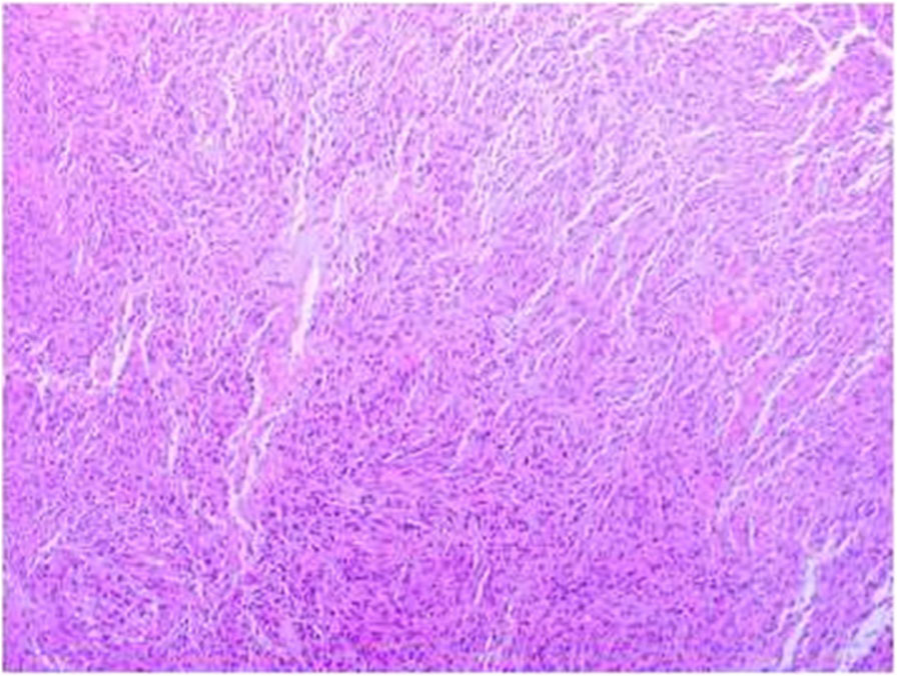
Histopathology. Biopsy results demonstrated spindle cell sarcoma with bleeding and necrosis


Given the above findings, the patient underwent right thoracic exploration and biopsy. Upon surgical inspection, we found a massive lobulated and friable tumour in the pericardium with severe bleeding (Fig. 4). There were extensive metastatic tumours with severe bleeding implanted on the right chest wall and the right lung. Intraoperative transoesophageal echocardiography clearly showed right atrial rupture with pseudoaneurysm formation (Fig. 5, video 1, video 2). Biopsy results demonstrated spindle cell sarcoma with bleeding and necrosis (Fig. 6). The patient was managed with conservative treatment. She died in April 2020.

**Additional file 1.** Movie S1. Intraoperative transoesophageal echocardiography clearly showed right atrium rupture with pseudoaneurysm formation


**Additional file 2.** Movie S2. Intraoperative transoesophageal echocardiography clearly showed right atrium rupture with pseudoaneurysm formation

## Discussion

Primary cardiac spindle cell sarcoma is an extremely rare tumour with a poor prognosis. Only a limited number of cases have been reported in the literature. Atrial rupture caused by spindle cell sarcoma has only been reported once [5]. Pseudoaneurysms caused by cardiac spindle cell sarcoma have never been reported worldwide. In this case, we described spindle cell sarcoma with a very high degree of malignancy causing right atrium rupture with pseudoaneurysm formation.

Spindle cell sarcomas are difficult to diagnose. First, the incidence of primary heart tumours is extremely low, with an incidence of approximately 0.017 to 0.019%, with 75% benign tumours and 25% malignancies. Most spindle cell sarcomas are initially diagnosed as myxoma [3, 6]. Second, there are no invasive criteria for the diagnosis of spindle cell sarcoma. Transthoracic echocardiography is the primary imaging modality for the initial diagnosis of intracardiac masses. However, its effectiveness can be limited by patient habitus and operator experience [7]. Transoesophageal echocardiography (TEE) also provides limited soft-tissue characterization and visualization of the mediastinum. The use of CT and CMR imaging to identify benign and malignant tumours remains a challenge. Therefore, malignant spindle cell sarcoma may be diagnosed only after surgical resection. Third, unlike other cancers, spindle cell sarcoma of the heart often occurs in young people aged 20 years to 50 years. A cardiac mass detected on TTE, CT or CMR usually fails to receive the attention of doctors. In our case, the patient, aged 32 years, first went to the hospital with very obvious clinical symptoms. CT and TTE revealed only pericardial effusion, pleural effusion and no mass in the heart. Repeated exfoliative cytology detection never revealed cancer cells. During tumour progression, the patient underwent repeated CT and CMR. Finally, the patient was definitively diagnosed with spindle cell sarcoma in the right atrium by surgery and histology. In conclusion, an atypical presentation of a cardiac mass on echocardiography, MRI, or CT angiography plus a rapidly progressive and recurrent clinical symptom should alert the physician to the possibility of a more aggressive or malignant disease.

Several treatments have been reported for cardiac spindle cell sarcoma, but the effect is poor. Surgical resection with negative margins has been recommended as the gold standard of treatment so far [8]. Nevertheless, the mean survival of patients was 3 months to 1 year because of incomplete tumour resection and malignancy recurrence [2, 9]. Chemotherapy and radiation therapy have limited benefits [10]. Heart transplantation may be an option for patients with sarcomas when there is no evidence of metastases; however, it has been associated with the recurrence of malignancy and new tumours stimulated by immunosuppression [11]. The guidelines regarding heart transplantation as candidacy for malignancies are unclear [12, 13]. Total artificial heart implantation for spindle cell sarcoma was also reported to have poor outcomes due to the risk and complications [12]. In this case, the patient unfortunately lost any prolonged life therapy.

## Conclusion

Spindle cell sarcoma of the heart is extremely rare with a difficult diagnosis, limited treatments and a poor prognosis. Recurrent pericardial effusion and pleural effusion, especially in the nature of haemorrhagic effusion, and/or right atrial pseudoaneurysm shown on the transthoracic echocardiogram must be considered and highly suspected as malignancy by patients and physicians. If the diagnosis cannot be confirmed, histopathology should be performed as soon as possible to avoid losing the best treatment opportunity. Surgical resection remains the main therapy. Heart transplantation for cardiac malignancies is rarely reported, and the effect is uncertain. Echocardiography remains the most common and routine screening method, and histopathological testing is the definitive method.

## Data Availability

Data sharing is not applicable to this article, as no datasets were generated or analysed during the current study.

## Abbreviations

CT: Computed tomography
TTE: Three-dimensional transthoracic echocardiogram
TEE: Transoesophageal echocardiogram
RA: Right atrium
PA: Pseudoaneurysm
Thromb: Thrombosis
RV: Right ventricle
LA: Left atrium
LV: Left ventricle
